# Proteinuria Triggers Renal Lymphangiogenesis Prior to the Development of Interstitial Fibrosis

**DOI:** 10.1371/journal.pone.0050209

**Published:** 2012-11-26

**Authors:** Saleh Yazdani, Fariba Poosti, Andrea B. Kramer, Katarina Mirković, Arjan J. Kwakernaak, Menno Hovingh, Maartje C. J. Slagman, Klaas A. Sjollema, Martin H. de Borst, Gerjan Navis, Harry van Goor, Jacob van den Born

**Affiliations:** 1 Division of Nephrology, Department of Medicine, University Medical Center Groningen, University of Groningen, Groningen, The Netherlands; 2 Division of Pathology, Department of Pathology and Medical Biology, University Medical Center Groningen, University of Groningen, Groningen, The Netherlands; 3 Microscopy and Imaging Center, University Medical Center Groningen, University of Groningen, Groningen, The Netherlands; University of Louisville, United States of America

## Abstract

Proteinuria is an important cause of progressive tubulo-interstitial damage. Whether proteinuria could trigger a renal lymphangiogenic response has not been established. Moreover, the temporal relationship between development of fibrosis, inflammation and lymphangiogenesis in chronic progressive kidney disease is not clear yet. Therefore, we evaluated the time course of lymph vessel (LV) formation in relation to proteinuria and interstitial damage in a rat model of chronic unilateral adriamycin nephrosis. Proteinuria and kidneys were evaluated up to 30 weeks after induction of nephrosis. LVs were identified by podoplanin/VEGFR3 double staining. After 6 weeks proteinuria was well-established, without influx of interstitial macrophages and myofibroblasts, collagen deposition, osteopontin expression (tubular activation) or LV formation. At 12 weeks, a ∼3-fold increase in cortical LV density was found (p<0.001), gradually increasing over time. This corresponded with a significant increase in tubular osteopontin expression (p<0.01) and interstitial myofibroblast numbers (p<0.05), whereas collagen deposition and macrophage numbers were not yet increased. VEGF-C was mostly expressed by tubular cells rather than interstitial cells. Cultured tubular cells stimulated with FCS showed a dose-dependent increase in mRNA and protein expression of VEGF-C which was not observed by human albumin stimulation. We conclude that chronic proteinuria provoked lymphangiogenesis in temporal conjunction with tubular osteopontin expression and influx of myofibroblasts, that preceded interstitial fibrosis.

## Introduction

Proteinuria is a noticeable risk factor for development of chronic kidney disease (CKD) and its reduction has renoprotective effects, slowing down progression to end-stage renal disease (ESRD) [1]–[3]. Proteinuria is not only a marker of renal failure progression, but is directly involved in the pathogenesis of tubulointerstitial fibrosis in the kidney as well [4].

Proximal tubular epithelial cells, experimentally exposed to pathologically high concentrations of plasma proteins, display several biologic responses, including profibrogenic signalling, inflammation, apoptosis, production of reactive oxygen species and epithelial dedifferentiation, which ultimately contribute to tubulointerstitial fibrosis [5]–[8]. With progression of the disease, macrophages become gradually involved in lesions, and this enhances proteinuria-induced renal structural changes into macrophage-dependent interstitial fibrosis [9], [10]. Tubulointerstitial fibrosis is important in long-term renal prognosis since it determines renal function and predict the outcome better than any other histopathological finding [11], [12]. The extent of proteinuria-induced tubulointerstitial changes limits the efficacy of antiproteinuric and renoprotective treatment with renin–angiotensin system (RAS) blockers [13], even when the changes are still within the pro-fibrotic range, highlighting the importance of tubulointerstitial damages in response to therapy in proteinuric patients. This warrants better exploration of the (pro-)fibrotic response to proteinuria.

Lymphatic vessels contribute to the drainage of extravasated proteins, excess fluid and macromolecules from interstitial tissue and return them to the blood circulation via the lymph, playing a crucial role in tissue fluid balance and homeostasis [14], [15]. They are also essential for immune defense by carrying antigens and antigen-presenting cells from the interstitium to the lymph nodes, a critical step for the development of an immune response [16]. The growth of lymphatic vessels (lymphangiogenesis) has been shown to be actively involved in adult tissues during various diseases such as inflammation, obesity, hypertension, tumour metastasis, organ transplantation and lymphedema [17], and very recently shown to be involved in development of fibrosis, at least in pulmonary fibrosis [18].

Renal lymphangiogenesis has been reported in transplanted kidneys [19], [20], and in nephropathies with interstitial fibrosis. Lymphangiogenesis generally correlated with the degree of tissue fibrosis rather than inflammation [21]. Recently, transforming growth factor-β (TGF-β) has been shown to induce VEGF-C expression in tubular epithelial cell which promote lymphangiogenesis [22]. Whether proteinuria could trigger a renal lymphangiogenic response has not been established. Studies up to now are mostly cross-sectional, and the temporal relationship between development of fibrosis, inflammation and lymphangiogenesis in chronic progressive kidney disease is not clear yet. To resolve these relationships, we performed a time-course study, up to 30 weeks, in adriamycin nephropathy in rats. We used a unilateral model to minimize possible effects of uremic condition and renal tissue oedema. Our study reveals that the formation of renal interstitial lymph vessels occurs after established proteinuria. The new lymphatic vessels are formed prior to collagen deposition and fibrosis, macrophage influx and in conjunction with the tubular osteopontin expression and VEGF-C production.

**Figure 1:**
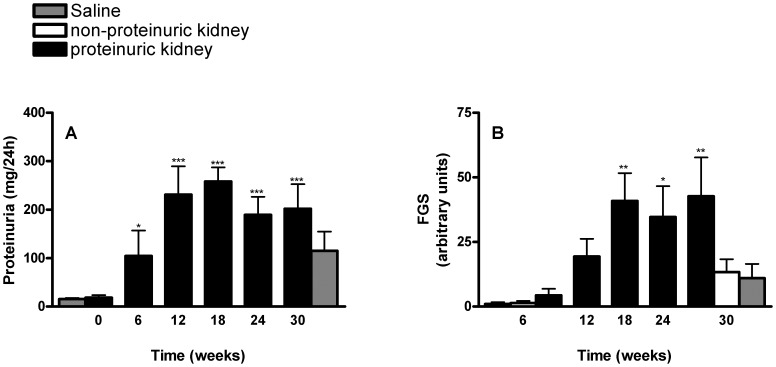
Development of proteinuria (A) and focal glomerulosclerosis (B) over time in unilateral Adriamycin-induced nephropathy. (*p<0.05, **p<0.01, ***p<0.001); (A) versus week 0; (B) versus week 6.

## Materials and Methods

### Animal Experimental Protocol

Unilateral adriamycin nephrosis was established in male Wister rats weighing 275–300 g, using a technique described previously [23]. Briefly, the left kidney was approached by means of an abdominal midline incision under isoflurane anesthesia (1.5% isoflurane in N_2_O/O_2_). An adjustable clamp was placed around the aorta above the left renal artery. Then, an injection of adriamycin (1.5 mg/kg) was performed in the tail vein. 12 minutes later, once adriamycin had been eliminated from the circulation [24], the clamp was removed. Rats in control group underwent the same surgical procedure with saline injection. Rats were placed in metabolic cages for 24-h urine collection bi-weekly, and proteinuria was determined in urine samples by a BNII third-generation nephelometer (Dade Behring, Mannheim, Germany). At different time points (week 6, 12, 18, 24, and 30) after the injection of adriamycin, 6 rats were sacrificed, and kidneys were harvested after perfusion with saline.

**Figure 2:**
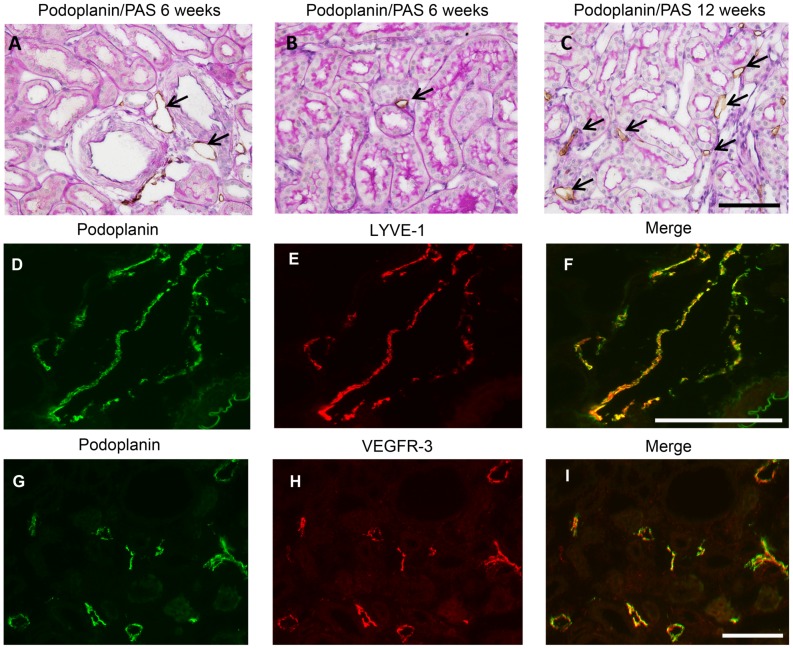
Identification of tubulo-interstitial lymph vessels. Podoplanin positive tubulo-interstitial vessel-like structures (indicated by black arrows) were present in the kidneys of saline-injected rats adjacent to large and middle-size arteries (A), and barely observed in cortical interstitial area, even in proteinuric kidneys at week 6 (B), however were clearly induced in the kidneys of adriamycin-injected rats at 12 weeks (C). Interstitial localization of these podoplanin-positive vessels becomes clear by the recognition of PAS-positive tubules. Double staining for podoplanin and LYVE-1 (D–F) and for podoplanin and VEGFR3 (G–I) confirmed interstitial podoplanin positive structures to be lymphatic endothelial cells, both in large and small lymphatic vessels. (Bar = 100 µm).

**Figure 3:**
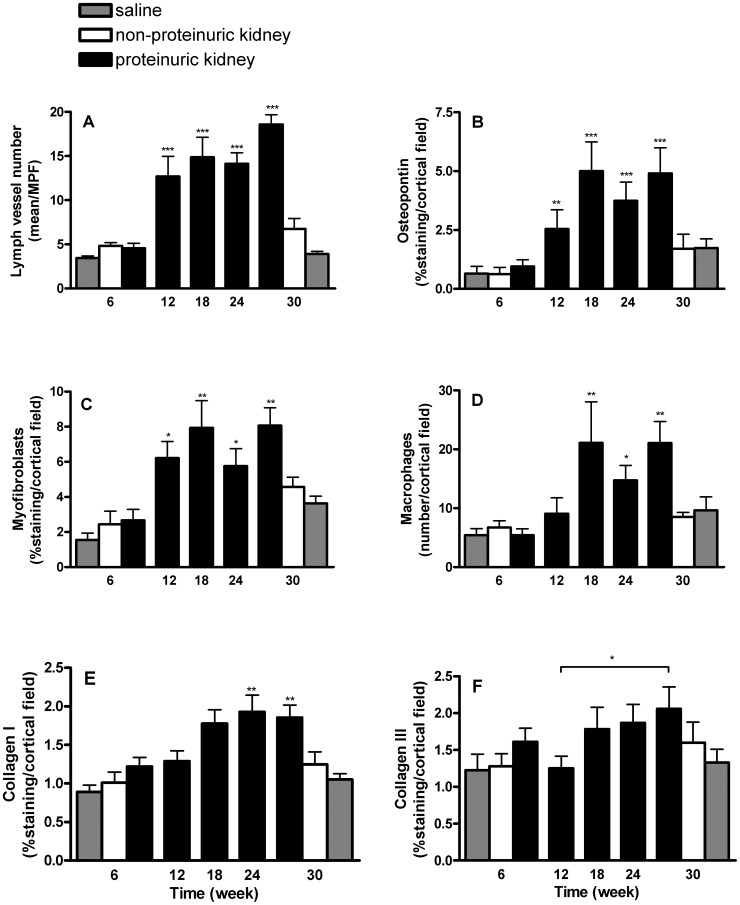
Quantitative immunohistochemical analysis of tubulo-interstitial damage markers in proteinuric, non-proteinuric and saline-injected rats over time. Lymphangiogenesis (A), measured as lymph vessel number per medium power field (MPF/200×); osteopontin staining (B), quantified as % positive staining per cortical field; myofibroblasts (C), quantified as % positive staining for α-smooth muscle-cell actin per cortical field; interstitial (ED-1)-positive macrophages (D), numbers per cortical field; interstitial collagen type I deposition (E), quantified as % staining per cortical field; and interstitial collagen type III deposition (F), quantified as % staining per cortical field (*p<0.05, **p<0.01, ***p<0.001 versus week 6, and for collagen III only week 12 versus week 30 was significant).

**Figure 4:**
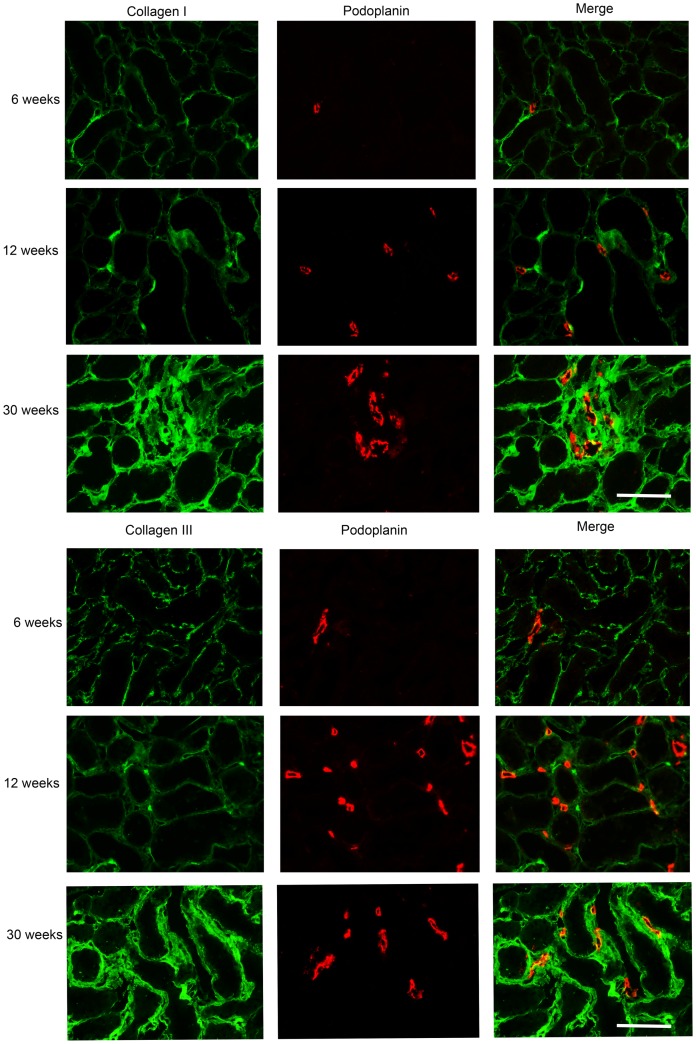
Interstitial lymph vessel induction precedes interstitial fibrosis. Double staining of podoplanin (lymph vessels) with the interstitial collagens types I and III shows the induction of lymph vessels from 12 weeks onwards, whereas interstitial fibrosis is only evident at week 30. (Bar = 100 µm).

In a separate bilateral adriamycin experiment, an intervention was done from week 6 to week 12 by the ACE inhibitor lisinopril (75 mg/l drinking water) in combination with low salt (0.05%) diet (n = 7 rats). Control adriamycin rats just received low salt diet from week 6 to week 12 (n = 6 rats). At week 12, 24-hours urine was collected in metabolic cages, animals were sacrificed and kidneys preserved for histology.

All experimental protocols for animal studies followed the national guide for the care and use of laboratory animals, and were approved by the local Animal Ethic Committee of the University of Groningen.

**Figure 5:**
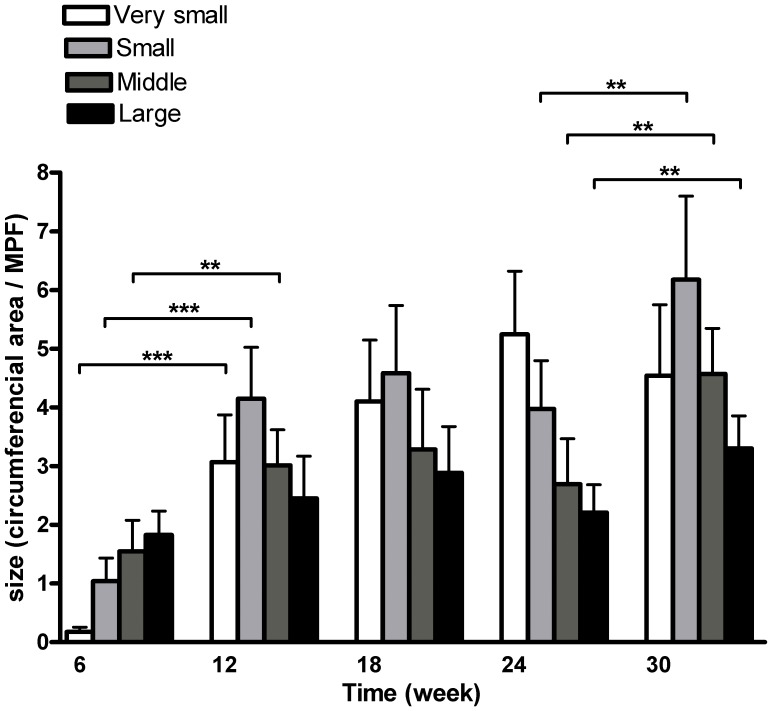
Quantification of surface area of all lymph vessels counted in proteinuric kidneys over time. All double positive lymph vessels have been divided into four categories based on their size. At 6 weeks, small, middle-size and large lymph vessels were present in close association with larger blood vessels, however there were almost no very small LV, indicating no lymph vessel formation. Over time, the number of very small LV significantly increased up to week 24. Besides, the number of small, middle-size and large LV increased between week 6–12 and/or between weeks 24–30, implying growth and enlargement of LV in these time frames. (**p<0.01; ***p<0.001).

### Immunohistochemistry and Immunofluorescence

Stainings were performed on both paraffin-embedded and cryo sections. Three-micron thick formalin-fixed paraffin sections were deparaffinized in xylene and rehydrated. Antigen retrieval was done for 10 min in citrate buffer, PH:6.0 or 10 mM Tris–1 mM EDTA buffer, pH: 9.0 in a microwave oven, and endogenous peroxidase activity was blocked with 0.3% hydrogen peroxide.

**Figure 6:**
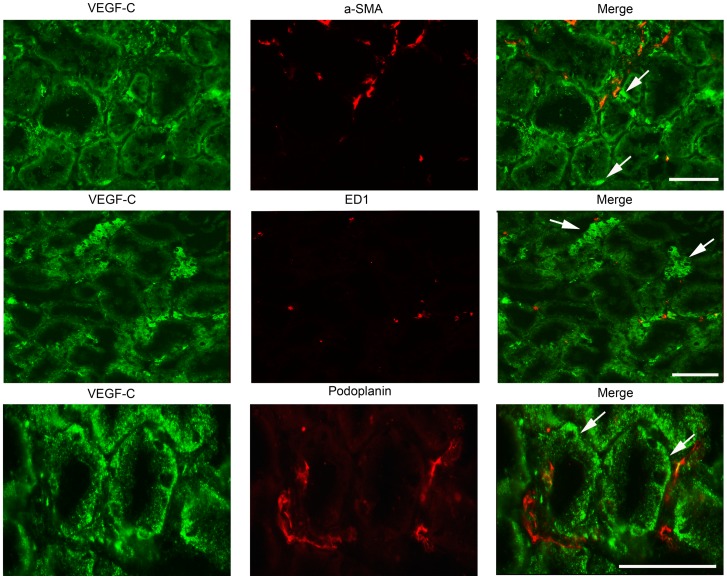
Double immunofluorescence staining for VEGF-C with α-SMA (myofibroblast), ED1 (macrophages), and with podoplanin (lymph vessels). Most podoplanin positive lymph vessels were observed close to tubules (mostly proximal) which showed VEGF-C expression at 12 weeks (arrows). After double staining for macrophages and myofibroblast, most of these interstitial cells were negative for VEGF-C. (Bar = 100 µm).

Four-micrometer thick frozen sections were fixed in acetone and treated with 0.03% hydrogen peroxide. Sections were pretreated with normal goat, rabbit or mouse serum, and subsequently incubated for 1 hr with the following primary antibodies: rabbit anti-LYVE-1 (Kind gift from Prof. David Jackson, John Radcliffe University Hospital, Oxford, UK), mouse anti-rat Podoplanin (Angio Bio, Del Mar, USA), Goat anti-mouse VEGFR3 (R&D system, Inc), mouse anti rat CD68 (Clone ED1, AbD Serotec, UK), rabbit anti-rat VEGF-C (RELIATech, GmbH, Germany), mouse anti-α-SMA (clone 1A4; Sigma), rabbit anti-collagen type I and rabbit anti-collagen type III (both from Biogenesis, Poole, UK). Binding of primary antibodies was detected by incubating the sections for 30 min with secondary antibodies diluted in PBS +1% normal rat serum: HRP-conjugated rabbit anti-mouse, biotinylated rabbit anti-mouse, FITC-conjugated rabbit anti-mouse, HRP-conjugated rabbit anti-goat, HRP and FITC-conjugated goat anti-rabbit and goat-anti mouse (all from DAKO, Belgium). As negative controls, the primary antibodies were omitted and replaced by PBS with normal rat serum. HRP and BIOT activity were visualized using the TSATM Tetramethylrhodamine System (PerkinElmer LAS Inc., USA) and FITC-conjugated streptavidin (Invitrogen) respectively. DAPI (Vector Laboratories, Inc) was used to stain nuclei. For paraffin sections, bound antibodies were visualized by the peroxidase substrate 3,3′-diaminobenzidine (DAB) (Sigma-Aldrich, USA) then counterstained with Periodic Acid Schiff (PAS).

**Figure 7:**
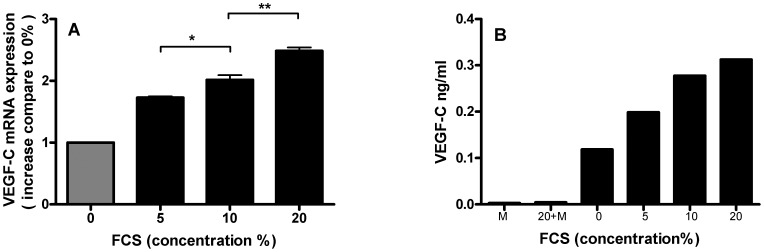
Human proximal tubular epithelial cells (HK-2) express VEGF-C upon serum stimulation in a dose-dependent fashion. After incubation of HK-2 cells by fetal calf serum, a dose-dependent induction of VEGF-C was observed both at mRNA level by RT-qPCR (A) and at protein level measured by ELISA in culture supernatant. mRNA induction (A) is expressed as increase in VEGF-C mRNA message compared to cells cultured in the absence of fetal calf serum. Graph represents three independent experiments performed in duplo. The VEGF-C ELISA (B) have been performed twice in duplo. Graph shows one representative example, with bars being the mean of duplicates. M (cell culture medium) and 20+M (20% FCS in medium without incubation with cells) were measured as controls (B). (*p<0.05; **p<0.01).

### Renal Histomorphology

Focal glomerulosclerosis (FGS), interstitial macrophages, myofibroblasts, osteopontin, collagen I and III were measured as described previously [23]. Briefly, interstitial macrophages (ED1 positive), myofibroblasts (α-SMA positive), osteopontin, collagen I and III immunostainings were evaluated in 50 cortical tubulo-interstitial images per slide using computerized image analysis (Advanced QUIPS, Leica Imaging Systems, Cambridge, UK) on blinded sections. The sections were semi-quantitatively scored for focal glomerulosclerosis in a blinded fashion by determining the level of mesangial expansion and focal adhesion in each quadrant in a glomerulus and expressed on a scale from 0 to 4 [25]. In total, 50 glomeruli per kidney were analyzed, and the total FGS score was calculated by multiplying the score by the percentage of glomeruli with the same FGS score. The sum of these scores gives the total FGS score with a maximum of 400.

**Figure 8:**
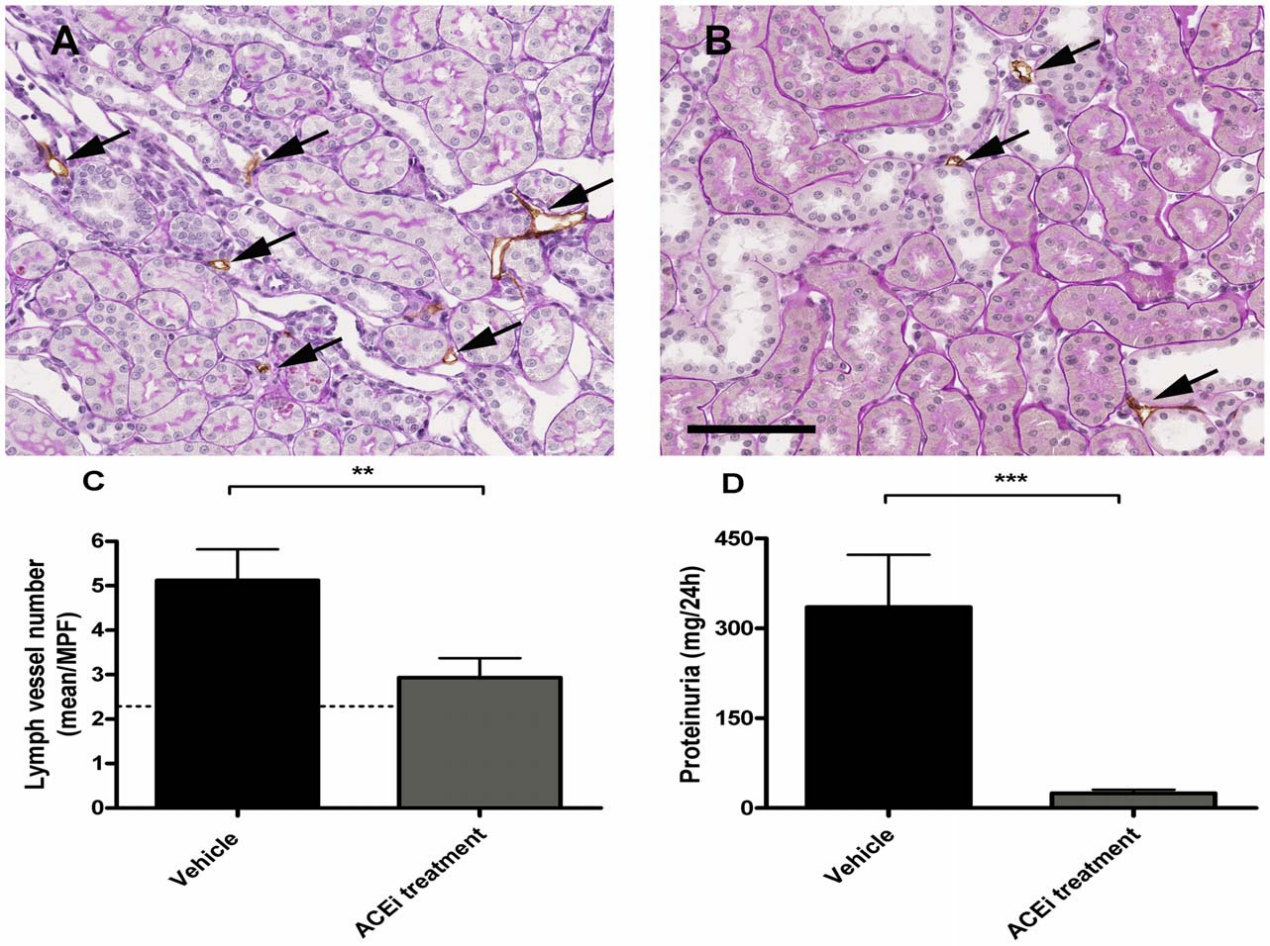
Antiproteinuric intervention strongly prevents renal lymphangiogenesis. Lymphatic vessel number was significantly higher in the kidneys of proteinuric rats than in rats receiving anti-proteinuric treatment. Representative images of interstitial lymphatic vessels (arrows) in the kidney of proteinuric (A) compared to treated animals (B). Quantification showed a significant reduction of lymph vessel number upon anti-proteinuric treatment (C), whereas efficacy of ACE inhibition on proteinuria is shown in D. Dashed line shows the mean number of cortical lymphatic vessels in kidneys of healthy rats. (Bar = 100 µm, **p<0.01, ***p<0.001).

### Double Immunofluorescence Protocol for Lymph Vessel Identification and Quantification

For identification of lymph vessels we applied a double-immunofluorescence protocol, using two primary antibodies against podoplanin and VEGFR3. After acetone fixation, frozen sections were blocked by 0.03% H2O2, followed by 20 minute incubating with appropriate normal serum. Then, sections were treated with mouse anti-rat podoplanin and goat anti-mouse VEGFR3 primary antibodies for 1 hr in room temperature. After washing three times with PBS (Phosphate buffered saline), FITC and HRP-conjugated secondary antibodies applied for 30 minutes to give each marker different colors. After staining, the sections were scanned using a Tissue FAXS acquisition system (Tissue Gnostics, Austria) based on a Zeiss Axio Observer Z1 (Zeiss, Germany). Microscopic fluorescence imaging was performed at the UMCG Microscopy and Imaging Center (UMIC), which is supported by the Netherlands Organisation for Health Research and Development (ZonMW grant 40-00506-98-9021). Double stained vessel structures which were positive for both markers (merged colors) counted as lymph vessels and measured by using a homemade macro on ImageJ 1.41 (Rasband, W.S., U.S. National Institutes of Health). A total of 30 fields per kidney cortex were evaluated and the lymphatic vessel density (LVD) was quantified as number of double positive (Podoplanin+/VEGFR3+), interstitial vascular profiles per medium-power field (20× objective).

The surface area of lymph vessels were also evaluated using Image J program, and lymph vessel size was differentiated into four size categories, namely: very small, small, medium- sized and larger lymph vessels.

### Tubular Epithelial Cell Culture

Human HK-2 cells (proximal tubular epithelial cell line) from American Type Culture Collection (ATCC, Manassas, VA, USA) were cultured in Dulbecco’s Modified Eagle’s Medium (DMEM) and Ham’s F-12 medium (Invitrogen-Gibco), supplemented with Insulin (5 µg/ml), Transferrin (5 µg/ml), Selenium (5 ng/ml), hydrocortison (36 ng/ml), Epidermal Growth Factor (EGF) (10 ng/ml), penicillin (50 U/ml) and streptomycin (50 µg/ml). The cells were cultured in 12-well plates until 90% confluency, then starved for two days without EGF. We stimulated the cells afterwards with different concentrations of FCS (Fetal Calf Serum, from Sigma-Aldrich), 5, 10 and 20% for 24 hours. Supernatant was collected for ELISA measurement, cells were washed with PBS (phosphate-buffered saline), and RNA was isolated for RT-PCR.

### RNA Extraction and Real-Time PCR

Total RNA was extracted using RNeasy Micro kit (Qiagen), and cDNA was synthesized using QuantiTect Reverse Transcriptase Kit (Qiagen) according to the manufacturer guidelines. The VEGF-C primer was QuantiTect Primer Assay (Qiagen, QT00175301), and as housekeeping gene, Glyceraldehyde 3-phosphate dehydrogenase (GAPDH) was used. Real-time PCR was performed using CFX384™ Real-Time System (Bio Rad) with SYBR Green assay (SensiMix™^,^ Bioline). Mean Ct values of samples were normalized to the GAPDH value (ΔCt), and relative results were expressed as 2^−ΔCt^.

### VEGF-C ELISA Measurement

The level of VEGF-C protein secreted into cell culture supernatant was measured by Human VEGF-C Immunoassay kit (R&D Systems, Wiesbaden-Nordernstadt, Germany), according to manufacturer’s instructions. Supernatant was collected from HK-2 cells stimulated by FCS. Medium alone, and medium +20% FCS were used as controls. Three individual experiments in duplicate were carried out.

### Statistical Analysis

Statistical analyses were performed using SPSS 16.0 (SPSS Inc., Chicago, IL) and figures were made using GraphPad Prism 5.0 (GraphPad Software Inc, La Jolla, CA). Differences among groups were tested using two way ANOVA with Tukey *post-hoc* analysis. Similar test was performed for non-parametric values after log transformation. Since many kidneys had FGS score of zero, the difference was tested with Kruskal-Wallis test with Dunn’s *post-hoc* analysis. P<0.05 was considered statistically significant.

## Results

### Clinical Parameters

Proteinuria was significantly increased in adriamycin-injected rats, compared to saline-injected controls. It was significantly increased as of 3 weeks (data not shown), and reached a plateau of ∼200–250 mg/24 h at twelve weeks after induction of nephrosis (Fig. 1A). FGS developed over time, with statistical difference from control as of 12 weeks (Fig. 1B). Rats in control groups developed a mild proteinuria over time, attributed to the normal process of aging. Serum creatinine concentrations, systolic blood pressures (SBP) and body weights were similar among all study groups (not shown).

### Lymph Vessel Number Increased in Conjunction with Tubular Activation but before Interstitial Fibrosis

Kidneys from adriamycin-injected rats showed the development of podoplanin-positive cells and vessel-like structures in the cortical interstitium (Fig. 2B and C), which were not seen in saline-injected control rats. In control renal tissues podoplanin-positive lymph vessels were only seen in close conjunction with larger arteries, mostly in deeper cortical regions (Fig. 2A). This strongly suggested the development of a podoplanin-positive lymphatic network in the cortical tubulo-interstitial compartment over time in proteinuric adriamycin rats. To be sure that these podoplanin-positive cells and vessel-like structures resembled lymphatic endothelium, we performed double staining for podoplanin with two other well-established markers for lymphatic endothelium namely VEGFR3 and LYVE-1. Indeed, almost all (more than 95%) interstitial podoplanin-positive cells and vessels were also positive for VEGFR3 and LYVE-1 (Fig. 2D–I), proving them to be lymphatic endothelial cells. Fig. 3A shows the absence of a lymphangiogenic response at 6 weeks after adriamycin injection, although proteinuria was already established with values around ∼100 mg/24 h. At 6 weeks no tubular activation (evidenced by tubular osteopontin expression), no interstitial accumulation of α-SMA positive myofibroblasts or interstitial influx of ED-1 positive macrophages was found (Fig. 3B–D). At week 12 a clear increase in lymphatics was observed, significantly different from week 6 (Fig. 3A, p<0.001). Evaluation of other tubulo-interstitial parameters revealed tubular activation (Fig. 3B, osteopontin week 12 versus week 6, p<0.01). Interstitial myofibroblasts were significantly increased at week 12 compared to week 6 as well (Fig. 3C, p<0.03), but interstitial macrophages (Fig. 3D), and interstitial fibrosis, evidenced by the interstitial collagens type I and type III were not yet increased at week 12 (Fig. 3E and F). From week 12 to week 30 a further increase of lymph vessels was found (Fig. 3A). Along, tubular activation (osteopontin), and interstitial accumulation of myofibroblasts and macrophages was observed (Fig. 3B–D). At weeks 24 and 30 interstitial collagen I significantly increased compared to week 6 (Fig. 3E, both time points p<0.01). Collagen III did only show a significant increase at week 30 compare to weeks 12 (Fig. 3F). In the control groups all interstitial parameters showed minor non-significant increase related to ageing of the animals. (Fig. 3A–F). These temporal data support a tubulo-interstitial lymphangiogenic response secondary to proteinuria, in conjunction with tubular activation by osteopontin expression and myofibroblast accumulation, however preceding interstitial inflammation (macrophage influx) and interstitial fibrosis. These findings are exemplified in Fig. 4.

### Size Distribution of Lymphatic Vessels Showed the Growth of Newly Formed LV Over Time

We analyzed surface area of all quantified lymphatic vessels in the kidneys from all adriamycin-injected rats. Based on the size distribution we divided them into four categories as shown in Figure 5. The distribution at 6 weeks showed the presence of small, middle-size and large lymph vessels. These are the already existing vessels present in close conjunction with larger arteries. No very small lymphatic structures are found at week 6, indicating the absence of new lymph vessel formation at week 6. At week 12, a significant increase is found in very small, small and medium-sized lymph structures (varying from single cells to very small vessel structures), indicating the occurrence of lymphangiogenesis and some size growth/maturation of lymph vessels. Between week 12 and 24 lymphangiogenesis continues, based on a further increase in very small lymphatic structures. In this time frame however, no growth/maturation of small, middle-size and large lymph vessels occurred. In the last phase, from week 24 to week 30, lymphangiogenesis decreased, whereas growth/maturation significantly increased. We conclude that growth, i.e. widening of vessel diameter, occurred mainly between weeks 6–12 and 24–30, whereas new lymph vessel formation takes place from week 6–24.

### Lymphangiogenesis is Seen in Conjunction with VEGF-C Positive Tubular Cells

Since VEGF-C has been described as a major lymphangiogenic factor in renal pathologies [16], [17], we stained renal tissues from adriamycin-injected rats at 12 weeks for VEGF-C. Many proximal tubules clearly showed VEGF-C expression in a baso-lateral fashion (Fig. 6, left panel). Podoplanin-positive lymph vessels were observed in close conjunction with tubuli positive for VEGF-C (Fig. 6, lowest panel). VEGF-C expression was also observed in a number of interstitial cells. To identify these VEGF-C positive interstitial cells we performed various double stainings for VEGF-C with α-SMA (myofibroblasts), ED-1 (macrophages), NG2 (pericytes), FSP-1 (fibroblasts), or CD31 (PECAM, endothelial cells). Although low percentages of all these different cell types (all <5%) showed some double positivity with VEGF-C, the majority of these cells did not express VEGF-C (Fig. 6. as an example just ED1 (middle panel) and α-SMA (top panel) is shown). Thus, we were not able to identify the phenotype of most interstitial cells which showed VEGF-C positive staining. Since however, main VEGF-C expression was observed in tubular epithelial cells and also the newly formed lymph vessels were mainly found closely to VEGF-C positive tubules (Fig. 6), our data suggest tubular VEGF-C to be involved in the lymphangiogenesis response.

### Expression of VEGF-C by Tubular HK-2 Cell Stimulated with FCS in Dose-dependent Manner

Since we showed tubular activation (osteopontin) and tubular VEGF-C expression at week 12 in response to prolonged proteinuria, we decided to test in vitro the capability of tubular epithelial cells to express and secrete VEGF-C during serum stimulation. We thus incubated growth arrested HK-2 proximal tubular epithelial cells with an increasing dose of fetal calf serum. By qPCR we found a dose-dependent induction of VEGF-C (Fig. 7A). By ELISA we measured a dose-dependent increase of VEGF-C in the supernatant, whereas control media in the absence of cells was completely negative (Fig. 7B). These in vitro experiments show that proximal tubular epithelial cells are able to induce VEGF-C mRNA and secrete VEGF-C protein upon serum stimulation.

### Anti-proteinuric Intervention Prevented Tubulo-interstitial Lymphangiogenesis

To test whether the tubulo-interstitial lymphangiogenic response was indeed secondary to proteinuria, in a separate experiment we treated adriamycin nephrotic rats from week 6 to week 12 with an ACE-inhibitor lisinopril under low salt diet. Control adriamycin rats were just on low salt diet from week 6 to week 12. Interestingly, the lymphangiogenic response seen in the control adriamycin rats was almost completely blunted upon the anti-proteinuric treatment by adding the ACE inhibitor (Fig. 8). Efficacy of the treatment is shown by an almost complete normalization of urinary protein excretion in the low salt/ACE inhibitor group. This intervention study clearly shows that an effective reduction in proteinuria prevented the interstitial lymphangiogenic response.

## Discussion

In this report we show in a unilateral adriamycin model in the rat that early, established, proteinuria is not associated with lymphangiogenesis. However, chronic proteinuria by tubular activation triggers new lymph vessel formation in the kidney, concomitantly with a pro-fibrotic response and osteopontin expression, and prior to the development of interstitial fibrosis. Tubular expression of VEGF-C in close conjunction with newly formed lymph vessels, together with in vitro tubular cell-derived VEGF-C production upon serum stimulation, suggest a role for activated tubular cells in the induction of new lymph vessel formation, secondary to proteinuria. In line with this, antiproteinuric treatment by ACE inhibition significantly prevented renal new lymph vessel formation compared to non-treated proteinuric rats.

Recent studies have shown a considerable heterogeneity in the expression of endothelial markers of lymphatic vessels in different physiological and pathologic conditions [26]. Despite this, LYVE-1 (lymphatic vessel endothelial hyaluronan receptor 1), VEGFR-3 (vascular endothelial growth factor receptor 3), Prox1 (Prospero-related homeobox transcription factor 1), and Podoplanin are among the most useful markers for microscopic imaging of lymph vessel, however, none is an ideal choice. To avoid misleading interpretation, use of multiple markers for lymph vessel staining has been proposed [27]. Therefore, we performed an immunofluorescent double-staining protocol using VEGFR-3 and Podoplanin for visualization of lymphatic endothelium. In the cortex of normal human and rodent kidneys, lymphatic vessels are restricted to the peripheral adventitia of large and middle-size arteries and virtually absent in tubuloinstertitium, whereas rarely observed in the medulla [20], [21]. In our study, lymphatic vessels were indeed adjacent to arteries in control kidneys, while in proteinuric kidneys from 12 weeks onwards massive proliferation of lymphatic vessels was observed especially in cortical tubulointerstitium but not in medullary region, fully in agreement with the expression of the lymphangiogenic factors VEGF-C and osteopontin by proximal tubules in cortical regions.

Proteinuria is associated with the activation of several molecular pathways which ultimately lead to tubulointerstitial inflammation and fibrotic interstitial damage [28]. Several *in vivo* and *in vitro* studies consistently have shown that plasma protein-associated factors can provoke direct tubular activation, and can induce the synthesis of several cytokines and chemokines, like endothelin-1 (ET-1), monocyte chemoattractant protein-1 (MCP-1), IL-8 and RANTES (regulated upon activation normal T-cell expressed and secreted), which all recruit inflammatory cells to the site of injury [5], [8], [29], [30]. Of particular interest among them is osteopontin. In addition to its multi-functional properties, very recently Liersch R. *et al*. identified osteopontin as a new lymphangiogenic factor in melanoma cell line [31]. Although we did not prove this in our study, it could be very well that OPN plays a role in renal lymphangiogenesis in our model, which is subject of future studies.

Infiltration of mononuclear cells in the pathogenesis of progressive renal injury has been well documented. Macrophages are closely engaged in the production of several cytokines and growth factors which contribute to ongoing tissue damage [32]. Several other cell types such as dendritic cells, neutrophils, mast cells and fibroblasts have been shown to secrete major lymphangiogenic growth factors, such as VEGF-C and -D, in inflammation-induced lymphangiogenesis [33], [34]. However, the role of macrophages has been highlighted mostly. Emerging evidence suggests that macrophages participates in lymphangiogenesis via two ways: secreting lymphangiogenic growth factors, and transdifferentiation into lymphatic endothelial cell [35], [36]. In our present study interstitial macrophage numbers increased significantly by 18 weeks, while at 6 and 12 weeks there was no prominent infiltration of macrophages as compared to controls. However, at 12 weeks with no significant increase of macrophage number, lymph vessel density sharply increased. Also a double staining for VEGF-C with macrophages (ED-1) showed the large majority (>95%) of them are not positive for this growth factor, suggesting that ED-1 positive cells, at least in our model, are not the main cells for secreting VEGF-C, which is in line with recent report in unilateral ureteral obstruction (UUO) model [22]. This implies that lymphangiogenesis is an early response, before influx of macrophages and development of inflammation, at least in renal proteinuric conditions, indicating different mechanism(s) for new LV formation in this model from well-characterised inflammation-induced lymphangiogenesis.

Lymphangiogenesis has been also reported in the remnant kidney model of renal fibrosis in rat [37], and in human renal biopsies where lymph vessel number was striking in tubulointerstitial fibrotic areas [21]. In our experiment, lymphangiogenesis significantly increased at week 12 without increase in interstitial collagen I and III deposition. These findings provide strong evidence that fibrosis and lymphangiogenesis are not always closely interconnected events, and are more in line with a profibrotic signalling mechanism in inducing lymphangiogenesis. Conflicting reports about the relation of lymphangiogenesis, fibrosis and TGF-β have been published and TGF-β shown as an inhibitor of lymphatic endothelial cell proliferation [38]–[40]. Recently in the kidney UUO model, TGF-β has been proved to induce VEGF-C expression by tubular epithelial cell which promote lymphangiogenesis [22]. Upregulation of TGF-β1 has been reported in human Proximal Tubular Epithelial Cells (PTEC) stimulated by human albumin serum (HAS), however the amount of TGF-β1 production was dependent on cell line [41]. Since TGF-β1 is a strong inducer of myofibroblasts, and in our study interstitial α-SMA positive myofibroblasts followed the same kinetics as lymph vessel density, we speculate that VEGF-C induction by TGF-β1 in tubular epithelial cells could be the mechanism of lymphangiogenesis in our proteinuric model. However, some other TGF-β1-independent mechanisms, like osteopontin, could be involved as well.

We did not investigate in detail which urinary proteins could induce tubular VEGF-C. We showed that plasma proteins (in fetal calf serum) are able to induce VEGF-C expression. We however were unable to induce VEGF-C in tubular cells by purified human albumin (data not shown). In line with this observation, in human minimal change nephropathy biopsies from patients with a selective albuminuria, lymph vessel formation was not observed, whereas in unselective proteinuric glomerulopathies, such as IgA nephropathy and membranous glomerulopathy, interstitial lymph vessels were induced [21].We thus speculate that albumin-unrelated proteins induce VEGF-C in tubular cells. In addition, the amount of filtered plasma proteins and proteinuria, and the duration of proteinuria may be important for tubular activation and induction of VEGF-C, since significant LVs formation was observed at 12 weeks, when proteinuria had established and osteopontin, as a marker of tubular activation, increased. It could be postulated that time and duration of contact between filtered plasma protein with tubular epithelial cells is an important factor to activate these cells for VEGF-C production and also for osteopontin expression. This suggestion is strongly corroborated by our finding that upon effective intervention on proteinuria, lymph vessel formation was almost completely prevented.

Although VEGF-C is the best characterized growth factor inducing lymphangiogenesis, we cannot claim that this growth factor is the sole lymphangiogenic mediator in our model, as many other mediators has been shown to be involved in lymphangiogenesis. Besides an eventual role for tubular osteopontin, several growth factors and cytokines are ultrafiltered from plasma into tubular fluid in proteinuria which normally are restricted from transglomerular passage [12], and some of them like hepatocyte growth factor (HGF) and platelet-derived growth factor (PDGF- BB), have been reported to have a potential lymphangiogenic ability [42]. There are other possibilities for the mechanism of renal lymphangiogenesis by proteinuria. One of the key mechanisms in the pathogenesis of proteinuria-associated injury is nuclear factor-kB (NF-kB), which upon stimulation evoking several proinflammatory responses [43]. NF-kB also plays a role in molecular mechanism of inflammation-induced lymphangiogenesis [44], which provides some evidence of underlying possible mechanism in proteinuria-driven lymphangiogenesis.

Edema in renal interstitium is another consequence of proteinuria [45]. Disruption of renal lymphatic circulation was shown experimentally leads to retention of protein and fluid in interstitium, severe proteinuria, fibrosis and renal cell apoptosis which caused chronic renal failure [15], [46]. On the other hand, inducing lymphangiogenesis, by using recombinant VEGF-C, has been reported to profoundly decrease interstitial fluid in experimental lymphedema [47], suggesting a beneficial effect of provoking lymphangiogenesis in ameliorating tissue edema. In our main experiment, we used unilateral adriamycin-induced proteinuric model to largely exclude effects of edema on lymphangiogenesis. Besides, the unilateral adriamycin approach prevents development of a uremic condition in the animals, thereby excluding eventual effects of uremic toxins on lymphangiogenesis. In transplantation, some experiments showed that lymphangiogenesis is detrimental by promoting migration of antigen-presenting cells (APCs) to the draining lymph node and initiating immune responses. Blocking lymphangiogenesis increased graft survival and better outcome afterwards, at least in experimental cardiac, corneal and islet transplantation [48]–[50]. In contrast, in experimental chronic and acute skin inflammation models lymphangiogenesis was beneficial by promoting lymph flow and reducing edema, suggesting a promising therapeutic strategy [51], [52]. Whether blocking lymphangiogenesis during proteinuria would have been beneficial effects for interstitial changes is not investigated in the current study and subject for further research.

In conclusion, our study for the first time showed that proteinuria can trigger renal lymphangiogenesis before development of interstitial fibrosis. Moreover, our data suggest lymphangiogenesis is induced by proteinuria-driven tubular activation, osteopontin expression and/or VEGF-C synthesis. The degree and duration of proteinuria seems crucial to activate tubular epithelial cell for production of osteopontin and VEGF-C. Future lymphangiostatic intervention studies will reveal the possible renoprotective effect of targeting lymphangiogenesis in proteinuric disease and the relation between lymphangiogenesis and development of fibrosis.
